# Why sequence all eukaryotes?

**DOI:** 10.1073/pnas.2115636118

**Published:** 2022-01-18

**Authors:** Mark Blaxter, John M. Archibald, Anna K. Childers, Jonathan A. Coddington, Keith A. Crandall, Federica Di Palma, Richard Durbin, Scott V. Edwards, Jennifer A. M. Graves, Kevin J. Hackett, Neil Hall, Erich D. Jarvis, Rebecca N. Johnson, Elinor K. Karlsson, W. John Kress, Shigehiro Kuraku, Mara K. N. Lawniczak, Kerstin Lindblad-Toh, Jose V. Lopez, Nancy A. Moran, Gene E. Robinson, Oliver A. Ryder, Beth Shapiro, Pamela S. Soltis, Tandy Warnow, Guojie Zhang, Harris A. Lewin

**Affiliations:** ^a^Wellcome Sanger Institute, Hinxton, Cambridge CB10 1SA, United Kingdom;; ^b^Department of Biochemistry and Molecular Biology, Dalhousie University, Halifax, NS B3H 4H7, Canada;; ^c^Bee Research Laboratory, Agricultural Research Service, US Department of Agriculture (USDA), Beltsville, MD 20705;; ^d^Global Genome Initiative, National Museum of Natural History, Smithsonian Institution, Washington, DC 20560;; ^e^Computational Biology Institute, Department of Biostatistics and Bioinformatics, George Washington University, Washington, DC 20052;; ^f^Department of Invertebrate Zoology, Smithsonian Institution, Washington, DC 20013;; ^g^School of Biological Sciences, University of East Anglia, Norwich NR4 7TJ, United Kingdom;; ^h^Department of Genetics, University of Cambridge, Cambridge CB2 3EH, United Kingdom;; ^i^Department of Organismic and Evolutionary Biology, Harvard University, Cambridge, MA 02138;; ^j^Museum of Comparative Zoology, Harvard University, Cambridge, MA 02138;; ^k^School of Life Sciences, La Trobe University, Bundoora, VIC 751 23, Australia;; ^l^University of Canberra, Bruce, ACT 2617, Australia;; ^m^Crop Production and Protection, Office of National Programs, Agricultural Research Service, USDA, Beltsville, MD 20705;; ^n^Earlham Institute, Norwich, Norfolk NR4 7UZ, United Kingdom;; ^o^Laboratory of the Neurogenetics of Language, The Rockefeller University, New York, NY 10065;; ^p^Howard Hughes Medical Institute, Chevy Chase, MD 20815;; ^q^National Museum of Natural History, Smithsonian Institution, Washington, DC 20560;; ^r^Bioinformatics and Integrative Biology, University of Massachusetts Medical School, Worcester, MA 01605;; ^s^Broad Institute of MIT and Harvard, Cambridge, MA 02142;; ^t^Botany, National Museum of Natural History, Smithsonian Institution, Washington, DC 20013-7012;; ^u^Department of Genomics and Evolutionary Biology, National Institute of Genetics, Mishima, Shizuoka 411-8540, Japan;; ^v^Laboratory for Phyloinformatics, RIKEN Center for Biosystems Dynamics Research, Kobe, Hyogo 650-0047, Japan;; ^w^Science for Life Laboratory, Department of Medical Biochemistry and Microbiology, Uppsala University, Uppsala 751 23, Sweden;; ^x^Department of Biological Sciences, Halmos College of Arts and Sciences, Nova Southeastern University, Dania Beach, FL 33004;; ^y^Guy Harvey Oceanographic Center, Dania Beach, FL 33004;; ^z^Integrative Biology, University of Texas at Austin, Austin, TX 78712;; ^aa^Carl R. Woese Institute for Genomic Biology, University of Illinois at Urbana–Champaign, Urbana, IL 61801;; ^bb^Department of Entomology, University of Illinois at Urbana–Champaign, Urbana, IL 61801;; ^cc^Conservation Genetics, Division of Biology, San Diego Zoo Wildlife Alliance, Escondido, CA 92027;; ^dd^Department of Evolution, Behavior and Ecology, University of California, San Diego, La Jolla, CA 92039;; ^ee^Department of Ecology and Evolutionary Biology, University of California, Santa Cruz, CA 95064;; ^ff^Florida Museum of Natural History, University of Florida, Gainesville, FL 32611;; ^gg^Biodiversity Institute, University of Florida, Gainesville, FL 32611;; ^hh^Department of Computer Science, University of Illinois at Urbana–Champaign, Urbana, IL 61301;; ^ii^Villum Center for Biodiversity Genomics, Section for Ecology and Evolution, Department of Biology, University of Copenhagen, Copenhagen 2100, Denmark;; ^jj^China National Genebank, Beijing Genomics Institute–Shenzhen, Shenzhen 518083, China;; ^kk^Department of Evolution and Ecology, College of Biological Sciences, University of California, Davis, CA 95616;; ^ll^Department of Population Health and Reproduction, University of California, Davis, CA 95616

**Keywords:** genome, diversity, ecology, evolution, conservation

## Abstract

Life on Earth has evolved from initial simplicity to the astounding complexity we experience today. Bacteria and archaea have largely excelled in metabolic diversification, but eukaryotes additionally display abundant morphological innovation. How have these innovations come about and what constraints are there on the origins of novelty and the continuing maintenance of biodiversity on Earth? The history of life and the code for the working parts of cells and systems are written in the genome. The Earth BioGenome Project has proposed that the genomes of all extant, named eukaryotes—about 2 million species—should be sequenced to high quality to produce a digital library of life on Earth, beginning with strategic phylogenetic, ecological, and high-impact priorities. Here we discuss why we should sequence all eukaryotic species, not just a representative few scattered across the many branches of the tree of life. We suggest that many questions of evolutionary and ecological significance will only be addressable when whole-genome data representing divergences at all of the branchings in the tree of life or all species in natural ecosystems are available. We envisage that a genomic tree of life will foster understanding of the ongoing processes of speciation, adaptation, and organismal dependencies within entire ecosystems. These explorations will resolve long-standing problems in phylogenetics, evolution, ecology, conservation, agriculture, bioindustry, and medicine.

“… every scrap of biological diversity is priceless, to be learned and cherished, and never to be surrendered without a struggle.”

E. O. Wilson (1)

Humans have classified the organisms of the natural world into groups by form and utility. In *On the Parts of Animals*, Aristotle (2) considered both form and function of animal organs and systems to ascertain deeper relationships between kinds. The flowering of scientific classification in the 300 y since Linnaeus (3) has given universal names to about 2 million eukaryotic species (https://www.catalogueoflife.org/*). The Linnaean project is not yet finished, as estimates of true eukaryotic diversity predict over 8 million extant species (4). Elucidation of the processes of evolution and speciation since Darwin’s *The Origin of Species* (5) demonstrates the interconnectedness of all life. Molecular characters, and especially phylogenetic analyses of DNA sequence data, have revealed the outline of the tree of life, but many of the details are yet to be discovered (6). Each species is a unique evolutionary experiment, the daughter of an unbroken lineage of successful experiments. To date, much of comparative genomics has centered on deep analysis of a short roster of species, focused around *Homo sapiens*. While this work has revealed many of the details of our and other species’ functioning and evolutionary history, each new genome has brought new insights and it is clear that our knowledge is limited to a small part of life’s true diversity (7). If we had the genomes of all species, we could ask questions across all species: What genes are unique to each group, or ecosystem, or process? How do genes and genomes change over time and space? What are the rules of evolution on grand and local scales? What diversity do we not know, and cannot currently predict?

The answers to these questions represent a quest for omniscience in biology—a quest to understand nature by characterizing its very essence, the DNA that encodes the basic blueprint of every species on Earth. This is the goal of the Earth BioGenome Project (EBP), a global effort to sequence the genomes of all currently named eukaryotic species (8, 9). In this article, we address fundamental reasons for sequencing all species (10), and pose sets of questions that can be addressed with large numbers of eukaryotic genomes across all clades. A digital library of eukaryotic genomes will form the fundamental infrastructure for the future of biology, agriculture, medicine, synthetic biology, and biomaterials science—a foundational legacy that democratizes and enables the science of the future (Fig. 1).

**Fig. 1.**
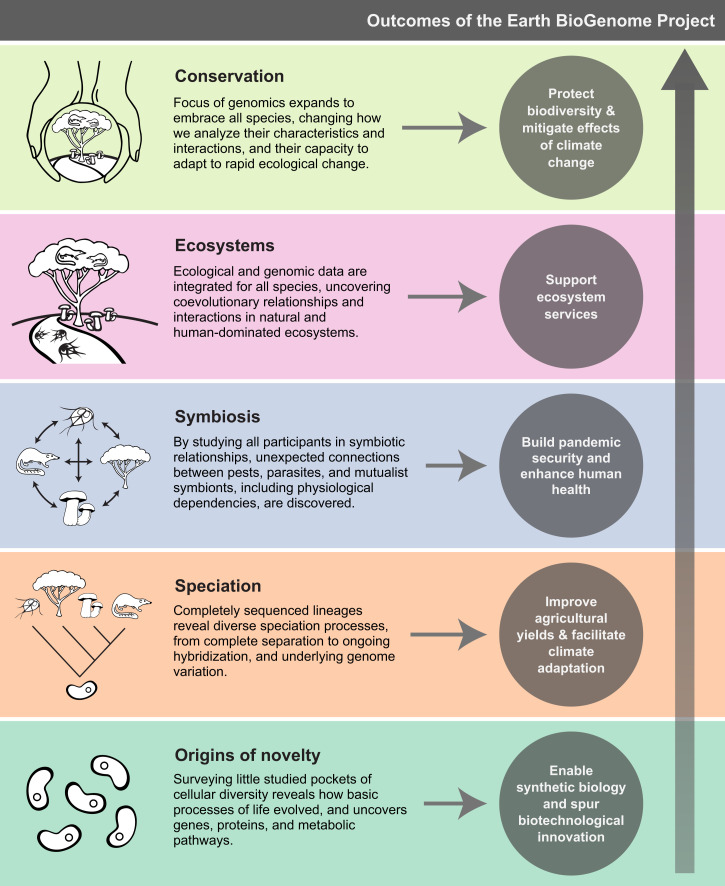
Outcomes of the Earth BioGenome Project. Sequencing all eukaryotic life on Earth will transform our understanding of how life evolved, help us build a sustainable future, provide biosecurity, and support an innovative bioeconomy. Here, we summarize the impact of these new genomes on basic research, which is described in detail in the text. Some practical benefits of this project are captured in the gray circles.

## Sequencing All Eukaryotic Life

The EBP and its affiliated projects^†^ propose the genome sequencing and annotation of all eukaryotic species (8). Each EBP-affiliated project has a different focus, be it geographic, ecosystem, or taxonomic, but they share the goal of producing high-quality reference genome assemblies. We aspire, whenever technically possible, to generate complete genome sequences that span each chromosome of the nuclear genome, and include all organellar genomes. The EBP aims to finish each species’ genome to a quality that will stand the tests of future science. This exacting standard is achievable using current long-read and long-range sequencing technologies, and using new bioinformatics toolkits designed to exploit the emergent properties of these exquisitely accurate data (11, 12).

The daunting task of sequencing ∼2 million species is made tractable by focused campaigns to address particular issues of interest. For example, the Vertebrate Genomes Project aims to sequence all 70,000 vertebrate species, but has started with reference genomes for each taxonomic order (12). The Darwin Tree of Life Project aims to sequence all 70,000 species in Britain and Ireland, and has started with a reference genome for each of the 4,200 taxonomic families represented (13). Other projects focus on species and ecosystems that are iconic for conservation, socially or scientifically important, or interesting because of their unique sets of adaptations. By close coordination under the EBP international network of networks, in particular developing platforms and processes that ensure synergy rather than conflict and promote diversity of target selection across life, the expertise and experience of thousands of scientists will be brought to bear on this “moonshot for biology.”

## Discovering the Trees of Life

Correct phylogenetic trees are essential data for the understanding of the origins and diversification of phenotypes. Dependent inferences will be unreliable if these trees are in error or unresolved. Should we even be thinking exclusively in terms of bifurcating trees (14, 15)? Hybrid origin of new plant species is common and hybridization, polyploidization, and horizontal gene transfer have also played major roles in diversification of other clades (16–19). Even without hybridization, gene trees across the genome commonly differ from the overarching species tree due to incomplete lineage sorting and the stochasticity of genetic drift (20). Existing sequence data have been used to build estimates of the tree of life at global and local scales, and this has resolved many questions. However, while sequence data are available in the International Nucleotide Sequence Database Collaboration databases (21) for 0.5 million species-level taxa,^‡^ very few sequences are available for most species and the overlap of homologous genes between species is low. Genome sequences have been used to explore the origins of eukaryotes and in revising the deep splits in protists, plants, and animals (22–25), but genomes, most in draft form, are available for only 0.4% of all species (9). In particular, the root of the tree and the placement of the many deep lineages of single-celled eukaryotes (the polyphyletic “protists”) remain unresolved (24). An accurate description of the tree of life will enable many deep questions about the evolution of eukaryotes to be definitively addressed (26), such as:

• What is the complete and true structure of the tree of eukaryotes?
• How is adaptive radiation reflected in genome structure and content, and are these radiations resolvable?
• How common and how important have hybridization and polyploidization been across the tree of life, and what signatures have ancient hybridization, polyploidization, and introgression left in genomes?
• When, where, and how do new genes arise? Are there predictable patterns of gene family origin and diversification in different lineages?
• How important is horizontal gene transfer in the evolution of eukaryotes?

Having all genomes will allow us to generate well-supported hypotheses of the origins and diversification of all branches of the eukaryotic tree, mapping reticulation events and timing of gene duplication, divergence, and loss. Complete genome sequences are more likely to contain information concerning nodes, processes, or events of interest. Complete sequences will permit rational choice of data to fit questions, for example choosing subsets with divergence rates that match the phylogenetic depth of the nodes being assessed. Full inventories of families of homologous genes, their protein sequences, and noncoding regulatory elements will highlight divergence and constraint in functional DNA, RNA, and amino acid residues, and inform bioengineering of new functions. It is clear that analyzing many thousands of whole genomes will require method development in orthology and phylogenetic inference toolkits.

## Defining the Origin of Eukaryotic Cells

Understanding the origin of the eukaryotic cell from prokaryotic precursors is one of the grand challenges of evolutionary biology. We still know relatively little about the antecedents of eukaryotic cellular complexity and the environmental and selective factors involved. Comparative cell biology and genomics tell us that the common ancestor of all known extant eukaryotes had a nucleus, temporospatially separated transcription and translation, a mitochondrion, linear chromosomes, meiotic sex, mitosis, an endomembrane and protein trafficking system, and a flagellum (27, 28). Homologs of many of the individual proteins involved in these diverse machineries and compartments can be found in bacteria and archaea—in particular the recently discovered Asgard archaea (29, 30)—but precisely how, when, and in what order they evolved is still far from clear. The origin of eukaryotes involved endosymbiotic assimilation of the bacterial ancestors of the mitochondrion and, in photosynthesizing lineages, the plastid (31, 32). This is analogous to many extant mutualist symbiotic systems, where a smaller partner, prokaryotic or eukaryotic, exists engulfed and reliant on a larger eukaryotic host. Fundamental questions about the evolutionary origin of eukaryotic cells can be addressed with a complete set of eukaryotic genomes, such as:

• What genes were present in the ancestor of all extant eukaryotes?
• Did eukaryotic cellular complexity evolve in a stepwise fashion or all at once?
• To what extent do present-day symbiotic processes model those acting at the origin of eukaryotes?
• What genomic processes limit and promote the horizontal spread of photosynthesis by secondary and tertiary endosymbioses?

Transformative insights into the origin of the eukaryotic cell are most likely to come from sequences of currently unknown pockets of cellular diversity within the archaeal and microbial eukaryotic domains, recognizing that key intermediates may now be extinct. With these data, generation of a highly resolved eukaryotic tree of life will underpin analyses of the origins and diversification of genes in the core eukaryotic toolkit and in organellar function.

## Tracking Genomic Changes in Symbiosis

Symbiosis is a signature of eukaryotic life, generating ecological diversity and organismal complexity across eukaryotic lineages. Primary productivity on land is based on mutualistic symbioses between vascular plants and root-colonizing microorganisms (33). Insect trophic niches are defined by acquisitions of beneficial microbial symbionts (34, 35). Mutualistic partners often delegate responsibility for essential processes to one another, enforcing codependence even in the face of barriers imposed by immune and antipredation systems (36). Species exist with a microbiome that aids in food digestion and other processes, and the genomes of the microbiome may be of relevance to understanding host physiology. Not all symbioses are mutualistic, and nearly one-third of described eukaryotic species are parasites (exploitative symbionts) (37). Parasites generate strong selection pressures on hosts and thus promote the maintenance of sex and the evolution of innate and adaptive immunity. Recognition of the ubiquity and fundamental ecological impact of symbioses is recent and largely stems from genomic data, which have shed light on symbiont origins, genome streamlining, and endosymbiotic transfer of genes to hosts (38). The big questions on symbioses that can be answered with genomes of all species include:

• Should hosts and their microbiomes be considered as hologenomic superorganisms?
• Are there common themes in the evolution of parasitic and mutualistic symbiont genomes?
• What are the range and diversity of mechanisms at the gene and molecular levels that enable symbioses?
• How do symbioses that are beneficial but not obligatory impact the evolution of both partners?
• How common is symbiotic gene transfer, and what legacies have vanished symbionts contributed to current-day organismal function?
• How much does coevolution between larger host organisms and their microbiomes drive diversification?

Access to the genomes of symbiotic partners of all eukaryotic species will enable discovery of novel genes and regulatory mechanisms that underlie the integration of distinct organisms, the opening of new ecological niches, and the promotion of species diversification. Understanding the relative evolutionary trajectories of hosts and their smaller partners will deliver understanding of the necessary linkage between these organisms, and may lead to directed enhancement of symbioses that benefit society or the environment. Understanding the essential physiological dependencies of pests and parasites and their mutualistic symbionts may also lead to novel intervention strategies.

## Decrypting Chromosome Evolution

Most bacteria and archaea have single, circular genomes and are haploid, but the genomes of most eukaryotes are diploid and organized in linear chromosomes. Eukaryotic chromatin is folded into a three-dimensional (3D) conformation that is critical to gene expression and cell differentiation (39, 40). Chromosome numbers range from one to many hundreds, and some species have different chromosome complements in germline and somatic nuclei through programmed DNA elimination (41, 42). Eukaryotes protect the ends of their chromosomes, the telomeres, from unavoidable replication-associated erosion through the addition of diverse kinds of nongenomically templated DNA (43). Centromeric function assures correct segregation of chromosome sets to daughter cells based on specific sequence features or epigenetic signals (44). The partitioning of genes on different chromosomes also offers opportunity and constraint in the evolution of gene regulatory networks. Within many eukaryotic taxa both karyotypes and synteny are generally conserved (45), while in others rearrangement is rampant even on short evolutionary timescales (46). Important questions about chromosomes that can be addressed with complete sampling of eukaryotic genomes include:

• What were the likely karyotypes, 3D organizations, and synteny relationships of genomes at all the ancestral nodes of the eukaryotic tree?
• How are chromosome numbers stabilized in the formation of a new species?
• What constraints do mitosis and meiosis put on chromosome number and organization?
• Are chromosome rearrangements the cause of major transitions and adaptations in eukaryotic evolution?
• How did telomeres evolve and what is the significance of different telomere maintenance mechanisms?
• Does the kind of centromere (e.g., holocentric versus centromeric) condition genome evolution?
• What constraints does karyotype place on the evolution of gene regulatory networks?

By generating chromosome-scale genome assemblies across the eukaryotic tree of life the evolution of chromosome organization at all scales can be explicitly addressed, from analysis of conservation of local synteny to karyotypic evolution, and the dynamics of centromere and telomere repositioning. These data could support the development of novel synthetic genomes with engineered chromosomal stability and other behaviors for biomedical and industrial applications.

## Revealing the Deep Logic of Eukaryotic Gene Regulation

Every eukaryotic genome contains thousands of protein-coding genes and each has regulatory elements and circuits that orchestrate how and when those genes are used. Most of these elements (47, 48), including noncoding transcripts (short and long), promoters, enhancers, insulators, and the 3D structure (49, 50) of the genome itself, can be distinguished from nonfunctional DNA because their sequences are evolutionarily constrained (51). Vice versa, regions with an accelerated rate of change in particular lineages may be linked to new evolutionary adaptations (52). For individual species, a reference genome makes it possible to assay regulation in tissues and cells by measuring gene expression, mapping open chromatin, detecting histone modification states, detecting transcription factor binding, and determining the 3D organization of chromatin against the reference genome assembly (48, 53). Some regulatory networks evolve rapidly, while others are apparently strongly constrained (54). Using comparative genomics, functional annotations experimentally determined in one species can be mapped across to related species, providing a rapid, in-depth genome annotation of a whole clade (55). Specific questions that can be addressed include:

• What is the comprehensive list of all eukaryotic genes?
• What are the building blocks of eukaryotic genome regulation? How stable are they?
• How do new regulatory regimes arise and supplant existing systems?
• Can deep multispecies whole-genome alignments reveal new classes of conserved elements?
• What genomic features distinguish conserved regulatory networks?
• How do regulatory networks constrain chromosome and genome evolution?

The power to discern patterns of constraint and acceleration depends on the number of species compared, and how closely related they are. With genomes of all species, functional sequences can be mapped with more sensitivity and at far greater resolution (56, 57), and this functional variation can be connected to phenotypes (58). Only with high-quality genomes can we map long-range chromosome interactions with confidence, and disentangle the effects of duplication, mobile element insertion, and repeat accumulation in modifying preexisting gene regulation. It will be possible to map regulatory promoter regions of protein-coding genes and long noncoding RNA, duplications, repeats, and transposable element insertions that are a crucial source of innovations of gene regulation (12, 59, 60). A far deeper understanding of the regulatory genome, and how it functions, will be gained than would ever be possible if each species was considered in isolation.

## Probing the Diversity of Sexual Systems

Sexual reproduction is a deep common thread in eukaryotes. It likely evolved to permit efficient mixing of alleles at linked loci to colonize new niches and escape parasites and pathogens (61). Asexual lineages are threatened by fitness degradation through accumulation of deleterious mutations (Muller’s ratchet) and are short-lived in phylogenetic terms, and putatively ancient asexuals appear to undergo rare sexual reproduction (62, 63). Some protist groups, such as ciliates, have multiple equivalent mating types, but most multicellular organisms have two sexes. Production of differentiated haploid gametes (large eggs and small sperm) evolved independently multiple times, and parental investment in eggs or sperm has led to evolution of extreme sex differences in morphology, life-history strategy, and behavior. There is tremendous variety in the mechanisms determining sex, ranging from single-locus drivers and differentiated sex chromosomes through to epigenetic and environmental determination (64, 65). The heterotypic sex chromosomes are often rapidly evolving, and sex determination mechanisms are often invaded by non-Mendelian elements (66). Sex determination systems provide strong evidence for evolution in action, but must simultaneously ensure continuing function of the essential processes of reproduction. Specific questions on the evolution of sexual systems that can be addressed by complete sequencing of eukaryotic lineages include:

• How often has a male–female system evolved independently, and how have systems with multiple mating types evolved and been maintained?
• Do changes in the sex determination system drive speciation, and how is this reflected in the genome?
• In species with segregated germlines (ciliates and most animals), how is the germline specified and how is the germline genome maintained?
• How is chromatin diminution distributed across the tree of life, and how is it regulated?
• Are there viable alternatives to meiotic recombination (such as gene conversion) that would allow ancient asexuals to avoid Muller’s ratchet, or do ancient asexuals just have sex very rarely?

Reference genome sequences for all taxa would lay bare the dynamics of the genomic causes and consequences of the evolution of sex determination systems. With chromosomally complete sequences, the dynamics of change in the sex-restricted chromosome in heterogametic groups could be precisely defined. Knowledge of the sexual systems of pests and parasites could be used to design gene drives that eliminate or reduce populations.

## Exploring Diversity in the Genomics of Speciation

Although the reality of eukaryotic species is accepted, the process of speciation is still a matter of debate, and there are many cases where species boundaries are leaky or incomplete. The biological species concept posits stable reproductive isolation between sister taxa (67, 68). However, genomic data show that ongoing gene exchange via hybridization and introgression after taxonomically accepted speciation is common, if not ubiquitous, and can occur over unexpectedly long time spans since initial separation (69). Frequently, nominal species concepts hide multiple cryptic taxa, and many species have yet to be discovered and named. For these new taxa, genomic data may be critical in discovery and definition (70). From the perspective of genetics and genomics, species separation is a process, not an event. Even in the absence of hybridization, variation at some loci remains shared (incomplete lineage sorting) typically for thousands to millions of generations even after total separation (20). The genetics and genomics of speciation mechanisms range from single loci of large effect, through inversions that suppress recombination or generate Haldane’s rule effects on the heterogametic sex, to genomes that have fully diverged in allopatry. Specific questions on speciation that can be addressed by complete sequencing of eukaryotic lineages include:

• How diverse are the processes of speciation and how do they pattern genomes?
• What genomic signals distinguish incomplete lineage sorting, introgression, and hybridization?
• Are certain kinds of genes and gene networks more likely to be implicated in speciation?
• What roles does chromosome rearrangement play in speciation?
• Do different reproductive strategies drive different genomic structures that impact on speciation?
• How do macroevolutionary phenomena such as species radiations pattern the genome?
• Is future speciation predictable from current-day genomes?

By sampling the full spectrum of species distinctiveness from ongoing hybridization to complete separation, the EBP will provide an enormously rich dataset to address key questions about the diverse processes of speciation and the impacts of speciation on genome structure and content. Genomics will become part and parcel of species description. A complete set of sequenced eukaryotic genomes will allow us to gain a deeper appreciation of not just the diversity of species but also the diversity of speciation mechanisms. We will develop a much richer appreciation of the ways in which species are distinct from each other.

## Decoding the Genomics of Complex Traits

Deciphering the genetic basis of complex traits has been one of the most challenging problems in contemporary biology, agriculture, and medicine. Variation in complex traits is generated by environmental, genetic, and genotype-by-environment interactions under the control of many genes with a range of effect sizes (71). Genome-wide association studies using thousands of single-nucleotide polymorphisms have confirmed the genetic complexity of quantitative traits and in some cases identified the genes or regulatory elements responsible for the genetic component of heritability estimates. However, the identity and modes of action of loci underpinning most complex traits remain enigmatic, and the roles of epigenetic factors in regulating complex traits have only recently come into focus. Comparative genomics across multiple independent origins can be used to identify convergent evolution of complex traits among species (26). For example, vocal learning evolved independently in several bird and mammal lineages, with convergent changes in expression of several hundred genes implicated in human speech (72), and repeated origins of sociality in bees are associated with changes in gene regulation (73). Sequenced genomes from across the eukaryotic tree of life can thus serve as a powerful resource for addressing important questions about the genomic architecture of complex traits, such as:

• What is the genomic nature of morphological homology?
• What traits can be mapped with higher power across versus within species?
• Where traits are shared by disparate taxa, what are the relative contributions of genetic homology (i.e., traits generated by homologous genetic toolkits) versus convergence?
• What genomic features predict and likely underpin the physiological systems that drive core traits of interest to conservation, human health, agriculture, and bioprocessing?
• Can organismal responses to climate change or other environmental disturbances be predicted from their genomes?
• What genomic and genetic architectures produce plasticity in responses and thus resistance or malleability to environmental change?

As the wider program of the EBP is achieved, the number of informative, independent replications of traits of interest accessible to whole-genome comparison will multiply, and these comparisons will be powerful because of the uniform quality of the genome assemblies and annotations. To achieve this vision, existing large-scale, rich, and open trait databases will need to be enhanced, collating physiological, life-history, and anatomical metadata that can be analyzed in the context of contiguous chromosome-level genome assemblies. Whole-genome alignment across many species can isolate trait loci to likely nucleotide, regulatory, and structural variants. Reference genomes for all taxa will also open each and every species-variable trait of interest to high-throughput genetic analysis. Overall, this comparative genomic approach, with high-quality genomes, applied to thousands of specialized traits in thousands of species, will lead to a new understanding of genotype–phenotype relationships, and ultimately define the rules of life (26).

## Understanding Ecosystem Function, Stasis, and Change

Biological diversity is often quantified by numbers of species in communities and in geographic regions, but ecological complexity and functioning are driven by species interactions. Organisms can be identified and counted using genetic signatures (DNA barcodes, environmental DNA), an approach limited only by the completeness of the reference libraries with which the signatures are compared (74). The biosynthetic capabilities and metabolic dependencies of species determine their abiotic ranges and thus potential species interactions, including coevolutionary relationships (75). A combination of genomic and ecological data can more completely elucidate species interaction networks within natural and human-dominated ecosystems (76). Because the interplay among species in a community is dynamic, adding historical dimensions to genomic investigations allows prediction of how ecosystems and species interactions will respond as environments undergo rapid change (77). One current, major ecosystem challenge is the increasing introduction of invasive species that can degrade local ecosystem function. Complete identification of species in ecosystems, including bacteria and archaea, will allow several grand challenge questions to be addressed, including:

• What are the genomic signatures within and between species that drive long-term interactions in biological communities and ecosystems?
• What is the genomic basis of ecological resilience?
• Can damaged or lost ecosystems be restored using knowledge of all species in the healthy state?
• Can invasiveness be predicted from species genomes, and can we use genomics to mitigate the effects of these invasive species?

A digital library of eukaryotic life will provide an anchored source of reference sequences for DNA barcoding, metagenomic, environmental DNA, and ancient DNA approaches to large-scale, high-throughput monitoring, and biosurveillance of present and past ecosystems. A library of all genomes will allow any environmentally sampled DNA sequence to be assigned to its species and even population of origin. Taxon presence and abundance derived from sequence surveys can link to the physiology of species inferred from their genomes, and thus be transformed into assessments of ecosystem balance (78). Even with the genome sequence of one individual, the coalescent history recorded in the genome can be used to estimate ancestral population sizes and thus compare current interactions with those of past ecosystems. Genomic understanding will enhance understanding of community species composition in time and space.

## Building Genomics-Informed Conservation

Earth is currently experiencing a sixth mass extinction of species, caused by humans (79). Species extinction is largely driven by habitat loss, either directly through habitat destruction, including fragmentation, or indirectly via climate change. Biodiversity is critical for maintenance of the essential ecosystem services on which human society depends (80, 81). While biodiversity loss is a product of runaway anthropogenic degradation, our active conservation and expansion of biodiversity are also part of the solution to the climate crisis. At the single-species level, there are concerns for the current, past, and dynamics of change of the gene pools of species, and captive or directed breeding initiatives rely on assessment and avoidance of inbreeding. Ecosystem fragmentation isolates different taxa in different ways, and building back from degraded fragments requires understanding of effects across diversity. These pressing issues raise questions for all of society, and reference-quality genomes for all eukaryotes can be part of the answers.

• How can ecosystem genomics be deployed to promote conservation of unsurveyed diversity?
• Can genomics robustly infer extinction risk and routes to extinction prevention, or even deextinction, for diverse species?
• Can predictions of extinction risk estimated from genome sequencing be integrated across species in an ecosystem?
• Is conservation of nearly neutral genetic diversity as important as conservation of adaptively evolving loci?
• How can genetic resilience be promoted in an ever-changing world where adaptation is needed in a geologically very short time frame?

Sequencing life is an opportunity to help preserve life. We suggest that the sequencing of the genomes of all species will change how we understand and analyze their characteristics and interactions, their population structures, and their likely capacity to adapt to rapid ecological change (8). The genomes of novel potential crops and crop relatives, and of diverse species that synthesize novel bioactive compounds, can build a new value economy where biodiversity is inherently valued for its future potential in agriculture or medicine. Genomically informed rewilding and ecosystem restoration could transform our planet and promote human coexistence with a thriving natural world. The skills built in rescuing our planet could be deployed in terraforming others. This knowledge will be key to preservation of species and interventions that maintain balance within ecosystems, and will drive effective, data-driven ecosystem conservation (82).

## Inventing New Tools and Resources

Historically, genome sequencing and assembly have been skilled labors, each polished genome the product of years of human effort (83). This has to change, without compromising on quality. While already routine for small bacterial and viral genomes, it is only now becoming possible to generate near-complete and error-free genome assemblies for eukaryotes (11) at scale (12), and high-quality genome sequencing from single, small specimens is becoming possible (84). These genome references are still estimates of the true genome sequence of an individual, but are orders of magnitude more contiguous and have higher per-base accuracy than previous generations of assemblies (11, 60). Similarly, while the discovery of coding and other features in genomes is still not perfect, current methodologies are generating very highly credible gene sets for downstream analyses. Turning a genome into a functioning organism is still something only the machinery of a living cell can do, but tools for predicting physiology and phenotypes from genome sequences are maturing rapidly, and inference of function will be made more robust with more complete, high-quality genomes, enhancing our phenotypic predictive power. The challenges include:

• Can we reliably and affordably generate telomere-to-telomere assemblies for all species, even those that have very few cells or that have very large genomes (or both)?
• Can we generate highly accurate estimates of the transcriptionally active parts of a genome, and thus of the proteins encoded and biochemical pathways present?
• Can the tools for comparative genomics (annotation, alignment, orthology inference) be refactored to analyze hundreds to tens of thousands of genomes simultaneously?

We are quietly but unashamedly optimistic in our assessment of both the possibility and promise of the scale of genomics that is proposed. Sequencing strategies are being refined actively, such that high-quality genome assemblies can be derived from even single small specimens and good draft-quality genomes from single cells (85). Algorithms that fully exploit the information contained in long-read sequence data are already reducing the computational costs of assembly while improving quality (86, 87). The annotation challenge is being met by new approaches, for example by leveraging the comparison between multiple genomes to identify conserved (and thus likely functional) regions (57, 88). The postgenome analytic processes are being conquered by rapid heuristic tools and the application of new data models, and will spur development of new statistical methodologies. In the end, the EBP will only achieve its goals if our tools are up to the task, and in building a toolkit that works at scale for the first 100,000 genomes we will, we believe, deliver a toolkit that works for millions. These tools will also serve the explosion of postgenomic research we expect to nurture and support.

## Conclusion and Outlook

The availability of highly accurate and fully assembled and annotated genomes densely sampled from across the millions of species on Earth will transform biological understanding (Fig. 1). This library of all life will preserve for posterity the diversity and history of this planet’s biology. The genomes will be the core data from which the phylogeny of all life is inferred, including the complex reticulations that endosymbiosis, horizontal transfer, hybridization, and introgression have created. Complete genome assemblies enable a broader and more complete understanding of a species’ biology, contributing to a lessened risk of extinction. Within the unifying model of this phylogenetic network, the genomes and the genes they possess will enable understanding of regulatory networks and trait evolution, the dynamics of coevolution between genes and between species, the impact of changing environments on species and populations, the mechanistic link between genotypes and phenotypes, and the drivers of genome–environment interactions. These analyses, in turn, will enable biologists to better characterize fundamental evolutionary processes, from the nucleotide to the genome level, identifying processes active under different chromosomal architectures and gene interaction networks. These dramatic advances in understanding of both the wide sweep and the local details of genomic and organismal evolution will enable the inference of ancestral genomes and their traits, which will be transformative for understanding how life evolved on Earth, predicting future evolution, and inspiring bioengineering of organisms with beneficial traits using technologies such as CRISPR and whole-genome synthesis. This foundational library of information will change the economic and social growth of the future, fostering sustainable agriculture and new bioeconomies, accessing an expanded medical pharmacopoeia, and promoting societal equity and diversity through the lens of a deeply valued biodiversity.

## Please Join the Conversation

The big questions we have posed derive from our collective discussions, but we are aware—and indeed hope—that there will be additional major questions that others believe can be answered by sequencing and functionally annotating all eukaryotic genomes. We invite you to add questions to the roster, to widen the debate, and to, ultimately, fully realize the promise of biological understanding based on the complete genome sequence of all of Earth’s remarkable species.

## Data Availability

There are no data underlying this work.

## Footnotes

*Currently 2,005,428 species; from Species 2000 & ITIS (89).

^†^https://www.earthbiogenome.org/institutional-members and https://www.earthbiogenome.org/affiliated-project-networks.

^‡^Value from https://www.ncbi.nlm.nih.gov/Taxonomy/taxonomyhome.html/index.cgi?chapter=statistics&uncultured=hide&unspecified=hide; including sequences from strains and isolates without a formal species name increases the count to ∼1.4 million.
